# Anti-emetic Drugs for Prophylaxis of Postoperative Nausea and Vomiting After Craniotomy: An Updated Systematic Review and Network Meta-Analysis

**DOI:** 10.3389/fmed.2020.00040

**Published:** 2020-02-25

**Authors:** Yijing Chen, Jing Chang

**Affiliations:** ^1^Department of Health Care, First Affiliated Hospital of Zhengzhou University, Zhengzhou, China; ^2^Academy of Medical Science, Zhengzhou University, Zhengzhou, China

**Keywords:** postoperative nausea, postoperative vomiting, emesis, postoperative complications, neurosurgery, craniotomy

## Abstract

**Background:** There is uncertainty about the effect of antiemetic drugs (AED) for the prophylaxis of postoperative nausea and vomiting (PONV) after craniotomy. In this study, we assessed the effectiveness and safety of AED for PONV.

**Methods and Findings:** We searched online databases including the Cochrane Library, PubMed, Wiley, Elsevier Science Direct, Ovid LWW, and Springer for publications from 1985 to June 2018. Adults undergoing craniotomy with the prophylactic use of at least one AED were included. The primary outcomes were the incidence of postoperative nausea (PON) and postoperative vomiting (POV) during the first and second day. A total of 1,433 participants from 17 clinical trials were enrolled in this Network Meta-Analysis (NMA). Compared to placebo, ramosetron was the most effective treatment for PON 24 h after surgery (OR = 0.063, 95% Crl: 0.006–0.45), with a 69.2% probability. On the other hand, for POV, droperidol was the best treatment during the first 2 h with a 71.1% probability (OR = 0.029, 95% Crl: 0.003–0.25); while fosaprepitant was the most effective treatment at 0–24 h (OR = 0.027, 95% Crl: 0.007–0.094; 66.9% probability) and 0–48 h (OR = 0.036, 95% Crl: 0.006–0.18; 56.6% probability). Besides, ramosetron showed a significantly higher incidence of complete response (OR = 29. 95% Crl: 1.4–6.5e + 02), as well as lower requirement for rescue AED (OR = 0.022, 95% Crl: 0.001–0.2). Granisetron was associated with the lowest incidence of headache and excessive sedation.

**Conclusions:** Compared with placebo, ramosetron appears to be the best prophylactic treatment for PON 24 h after craniotomy, with higher complete responses. Fosaprepitant appears to be the most effective prophylaxis option for POV on the first 0–24 and 0–48 h. Both may be better applied in combination with perioperative dexamethasone. These findings may guide clinicians to provide improved pharmacological prophylaxis for PONV after craniotomy with fewer adverse effects.

## Introduction

Postoperative nausea and vomiting (PONV) are some of the most frequent postoperative complications, with incidence rates as high as 50% following craniotomy. In high-risk patients without prophylactic anti-emetic drugs (AED), the incidence of PONV can reach 80% (1). Previous research has shown that PONV increases the risk of additional complications post-craniotomy (2). Although PONV is not life-threatening, compared to major neurological complications such as hematomas, it can modify intracranial pressure and trigger more severe subsequent events. In addition, PONV frequently prolongs hospital stays and decreases patient satisfaction (1, 3). Risk factors for PONV after neurosurgery include gender and age; specifically, PONV is most prevalent in young female patients with a history of previous PONV (4). Abundant research has focused on lowering the incidence of PONV, particularly in high-risk patients undergoing craniotomy (5).

The latest guidelines for the management of PONV recommend the administration of prophylactic AED as part of a multimodal therapy in high-risk adults. Commonly used AED for PONV include 5-hydroxytryptamine (5-HT3) receptor antagonists, neurokinin-1 (NK-1) receptor antagonists, corticosteroids, butyrophenones, antihistamines, anticholinergics, phenothiazines, and other unclassified compounds (5). The effectiveness of these AED for the prevention of PONV has been well-documented (5, 6), but evidence-based data supporting prophylactic AED use in craniotomy patients is lacking. The most recent systematic review article on this topic was published in 2007, and there is currently insufficient direct and comparative treatment data to make evidence-based prophylactic treatment decisions regarding AED for PONV (2). In this NMA, we assess the effectiveness and safety of AED for postoperative nausea (PON) and postoperative vomiting (POV) at different postoperative time intervals.

## Methods

### Methodology

A Bayesian network meta-analysis was used. Network meta-analysis integrates data from direct and indirect comparison of trials, allowing the evaluation of different trials and treatments. Network meta-analysis also allows the ranking of treatments, and their efficacy or safety can be expressed as a percentage of the perfect intervention which is undoubtedly the best choice. Network meta-analysis thus provides more comprehensive information compared to traditional meta-analysis.

We performed a systematic literature review according to the Preferred Reporting Items for Systematic Reviews and Meta-Analyses (PRISMA) statement (7). The review protocol is available on the International Prospective Register of Systematic Reviews (PROSPERO), registration number: CRD42018092832. Relevant databases were searched, including Cochrane Library, PubMed, Wiley, Elsevier Science Direct, Ovid LWW, and Springer from inception to June 11, 2018. We searched for randomized controlled trials on the use of AED for PONV prophylaxis following craniotomy. We used terms such as “postoperative nausea and vomiting,” “craniotomy,” “postoperative nausea,” “postoperative vomiting,” and “postoperative emesis”; and explored related MeSH terms and keywords. The complete search strategy is outlined in Appendix 1. Two reviewers independently selected relevant studies by screening publication titles and abstracts (YC and JC). The full texts of the included articles were fully assessed. Disagreements were settled by consulting with a third reviewer.

### Patient Criteria

We included randomized controlled trials (RCT) on the effects of AED on PON and/or POV in comparison to either placebo or other AED. We included studies performed in adults of any gender undergoing supratentorial or infratentorial craniotomy, with the prophylactic use of at least one AED. Studies involving children were excluded due to the insufficient development of their metabolism and their higher incidence rate of postoperative emesis in comparison with adults (6). Trials where PONV incidence was not the primary outcome were excluded. We also excluded trials in which craniotomy was performed during wakefulness. For multiple trials from the same authors investigating the prophylactic effects of the same AED, we included only the most recent study. No such restriction was applied if different AED were used.

### Outcome Measures

The primary outcome was the incidence of PON, POV or both (PONV) during the first two postoperative days. PONV data was presented as the number of patients who experienced vomiting. The secondary outcomes were complete response, requirement for rescue AED, and the incidence of drug-related side effects. Complete responses were defined as patients without PONV and who did not require rescue AED. Adverse events included dizziness, headache, and excessive sedation.

In studies that presented outcomes as percentages, sample sizes were used to multiply those percentages. If data were presented as comprehensible graphs, data were estimated through algorithmic computation, as well as x- and y-axis line drawing (6). If the trial possessed multiple treatment arms with varying drug dosages, we included the treatment arm with the same drug and dosage regimen as other trials. If the study reported PONV in the absence of distinct nausea and emesis data, PONV was regarded as emesis.

According to the latest guidelines for PONV management (5), we extracted the following information: sex, age, duration of anesthesia, intraoperative and postoperative dexamethasone use, AED name and dosage, and clinical outcomes (adverse events).

### Risk of Bias Assessment

Risk of bias assessment was performed using the Cochrane collaboration's tool Rev Man 5.3. This software evaluates bias based on (a) selection bias: random sequence generation and allocation concealment; (b) performance bias: blinding of participants and personnel; (c) detection bias: blinding of outcome assessments; (d) attrition bias: incomplete outcome data; and (e) reporting bias: selective reporting and other bias.

### Data Synthesis

For each outcome, we used the random effects model with Bayesian approach to perform direct and indirect treatment comparisons. OR and 95% credible intervals (95% Crls) were calculated using the Markov Chain Monte Carlo method with GeMTC. We also performed a pair-wise meta-analysis and calculated pooled OR for direct comparison in order to assess consistency. *I*^2^ was used to assess heterogeneity. The node-splitting model was used to identify inconsistencies with the Bayesian *P-*value. Each AED outcome was explored based on the distribution of ranking probability and the surface under the cumulative ranking curves (SUCRA). These show the rank of every intervention based on their probability of effectiveness. Sensitivity analyses were performed by omitting studies with a high risk of bias, as well as those that did not use perioperative dexamethasone.

All data were analyzed in a Bayesian framework using the GeMTC R package online analysis system (https://gemtc.drugis.org/). PRISMA flow chart and risk of bias assessments were conducted using Review Manager 5.3 software.

## Evidence Synthesis

### Literature Search

**Figure 1:**
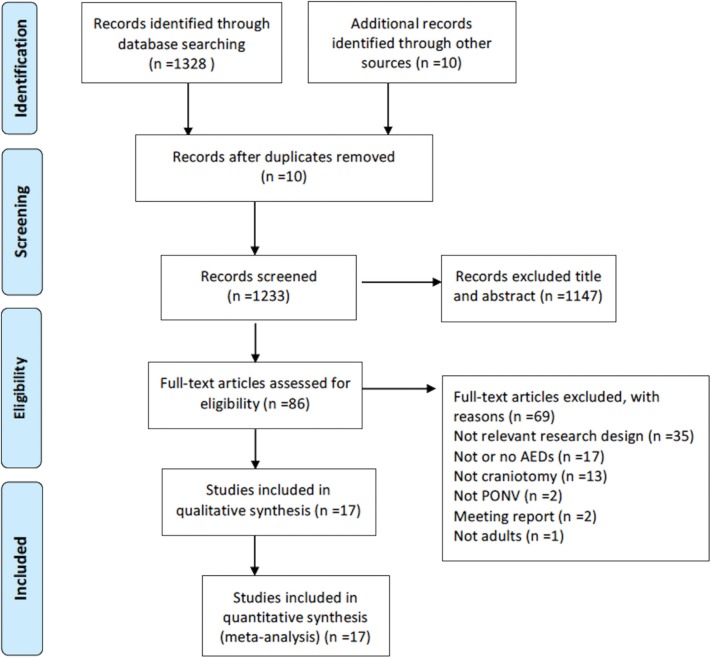
Flow diagram of the literature search.

A total of 1,233 unique citations were identified. After examining titles and abstracts, 86 potentially relevant articles were identified, and a full-text review of each article was performed (see Appendix for a comprehensive list of these studies). A final number of 17 trials with a total of 1,433 participants were enrolled in this NMA (8–24) (Figure 1). Fifteen trials assessed PON, whereas all 17 trails assessed POV. A complete description of the full-text review is shown in the supplementary data (Appendix 1).

### Characteristics of the Identified Trials

The characteristics of each clinical trial included in the NMA are listed in Table 1. Female participants accounted for 52% of patients, with age ranging between 32 and 63 years. Three studies reported no intraoperative or postoperative dexamethasone use (21, 22, 24). The AED investigated included 5-hydroxytryptamine (5-HT3) receptor antagonists (granisetron, ondansetron, ramosetron, and tropisetron), neurokinin-1 (NK-1) receptor antagonists (fosaprepitant, aprepitant), butyrophenones (droperidol), corticosteroids (dexamethasone), phenothiazines (metoclopramide), and other unclassified drugs (gabapentin). Dexamethasone was not directly included as an independent AED, as it was considered an adjuvant used in combination with other AED. In this NMA, five trials were single therapy (11, 20–22, 24) and 11 trials included a combination of AED with dexamethasone (8, 9, 12–19, 23). Two trials in which dexamethasone was used in <50% of the enrolled patients were classed as single therapy. Only one clinical trial performed triple therapy with multimodal AED (10). Four trials had three treatment arms (12, 14, 16, 20) whilst the other 13 were two-arm trials (8–11, 13, 15, 17–19, 21–24).

**Table 1:** Baseline characteristics of the studies included in the NMA.

**References**	**Total size**	**Region**	**Intervention**	**Control**	**Patient characters**	**Perioperative** **dexamethasone**	**Side effect**
Shobaki et al. (8)	40	Egypt	Granisetron 20 ug/kg	Placebo	Median age: Granisetron 56 year; Placebo 53 year Female (%): Granisetron 40%; Placebo 50% MAD (min): Granisetron 327; placebo 293	YES	Dizziness/ confusion/ extrapyramidal
Atsuta et al. (9)	186	Japan	Fosaprepitant 150 mg	Droperidol 1.25 mg	Median age: fosaprepitant 62 years; Droperidol 63 years Female (%): Fosaprepitant 60%; Droperidol 55% MAD (min) Fosaprepitant 342; Droperidol 353	YES	NR
Bergese et al. (10)	95	USA	Aprepitant 40 mg	Ondansetron 4 mg	Median age: Aprepitant 52 years; Ondansetron 51 years Female (%): Aprepitant 54%; Ondansetron 53% MAD (min): Aprepitant 308; Ondansetron 345	YES	NR
Pugh et al. (11)	60	England	Metoclopramide 10 mg	Ondansetron 8 mg	Median age: Metoclopramide 54 years; Ondansetron 51 years Female (%): Metoclopramide 57%; Ondansetron 67% MAD (min): Metoclopramide 196; Ondansetron180	YES	NR
Fabling et al. (12)	60	USA	Ondansetron 4 mg Droperidol 0.625 mg	Placebo	Median age: Ondansetron 52 years; Droperidol 45 years; Placebo 47 years Female (%): Ondansetron 60%; Droperidol 45%; Placebo 55% MAD (min): Ondansetron 281; Droperidol 312; Placebo 328	YES	sedation
Fabling et al. (13)	46	USA	Ondansetron 4 mg	Placebo	Median age: Ondansetron 53 years; Placebo 55 years Female (%): Ondansetron 52%; Placebo 48% MAD (min): Ondansetron 325; Placebo 282	YES	Pain sedation
Habib et al. (15)	104	USA	Aprepitant 40 mg	Ondansetron 4 mg	Median age: Aprepitant 51 years; Ondansetron 48 years Female (%): Aprepitant 55%; Ondansetron 57% Mean surgery duration (min): Aprepitant 180; Ondansetron 179	YES	Sedation headache
Jain et al. (16)	87	India	Ondansetron 4 mg Granisetron 1 mg	Placebo	Median age: Ondansetron 34 years; Granisetron 38; Placebo 34 years Female (%): Ondansetron 19%; Granisetron 27%; Placebo 43% MAD (min): Ondansetron 281; Granisetron 263; Placebo 288	YES	Headache dizziness confusion
Kathirvel et al. (17)	152	India	Ondansetron 4 mg	Placebo	Median age: Ondansetron 39 years; Placebo 37 years Female (%): Ondansetron 52%; Placebo 48% MAD (min): Ondansetron 274; Placebo 277	YES	Sedation/ confusion/ dizziness/ constipation/ extrapyramidal
Madenoglu et al. (18)	60	Turkey	Tropisetron 2 mg	Placebo	Median age: Tropisetron 45 years; Placebo 44 years Female (%): Tropisetron 60%; Placebo 43% MAD (min): Tropisetron 288; Placebo 347	YES	sedation
Misra et al. (19)	73	India	Gabapentin 600 mg	Placebo	Median age: Gabapentin 45 years; Placebo 44 years Female (%): Gabapentin 44%; Placebo 46% MAD (min): Gabapentin 400.8; Placebo 400.9	YES	NR
Ryu et al. (20)	160	South Korea	Ondansetron 4 mg Ondansetron 8 mg	Ramosetron 0.3 mg	Median age: Ondansetron^1^ 49 years; Ondansetron^2^ 48 years; Ramosetron 53 years Female (%): Ondansetron^1^ 66% ondansetron^2^ 57%; Ramosetron 69%	NO	Drowsiness dizziness itching
					MAD (min): Ondansetron^1^ 321 Ondansetron^2^ 368; Ramosetron 327		
Sinha et al. (21)	40	India	Ondansetron 4 mg	Placebo	Median age: Ondansetron 36 years; Placebo 33 years Female (%): Ondansetron 55%; Placebo 40% MAD (min): Ondansetron 368; Placebo 344	NO	Sedation/ confusion/ diarrhea/ constipation
Tsutsumi et al. (22)	64	Japan	Fosaprepitant 150 mg	Ondansetron 4 mg	Median age: fosaprepitant 62 years; Ondansetron 58 years Female (%): Fosaprepitant 53%; Ondansetron 66% MAD (min): Fosaprepitant 460; Ondansetron 513	NO	Pain
Wang et al. (23)	70	China	Granisetron 3 mg	Placebo	Median age: Granisetron 42 years; Placebo 39 years Female (%): Granisetron 51%; Placebo 54% MAD (min): Granisetron 368; Placebo 344	YES	NR
Wig et al. (24)	70	India	Ondansetron 4 mg	Placebo	Median age: Ondansetron 40 years; Placebo 40 years Female (%): Ondansetron 46%; Placebo 34% MAD (min): Ondansetron 269; Placebo 242	NO	NR
Gupta et al. (14)	75	India	Granisetron1 mg Ondansetron 4 mg	Placebo	Median age: Ondansetron 37 years; Granisetron 36; Placebo 37 years Female (%): Ondansetron 52%; Granisetron 52%; Placebo 48% MAD (min): Ondansetron 239; Granisetron 258; Placebo 246	YES	Sedation

*MAD, mean anesthesia duration; NR, not reported*.

1 *Ondansetron 4 mg*.

2 *Ondansetron 8 mg*.

### Risk of Bias

**Figure 2:**
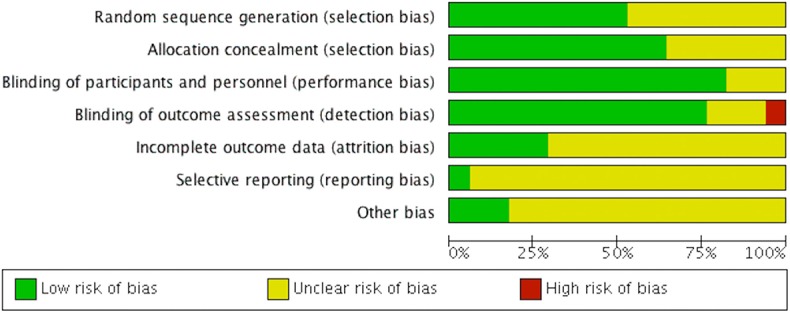
Risk of bias assessment.

Amongst the included RCT, 43% reported unclear allocation concealment, and 50% reported random sequence generation. Most of the included RCT had unclear or incomplete outcome data (70%). Unclear selective reporting risk of bias was found in nearly all RCT (95%), which may be related to the lack of availability of the study protocols (Figure 2).

### Effectiveness of Anti-emetic Drugs for Postoperative Nausea During the First 24 h

**Figure 3:**
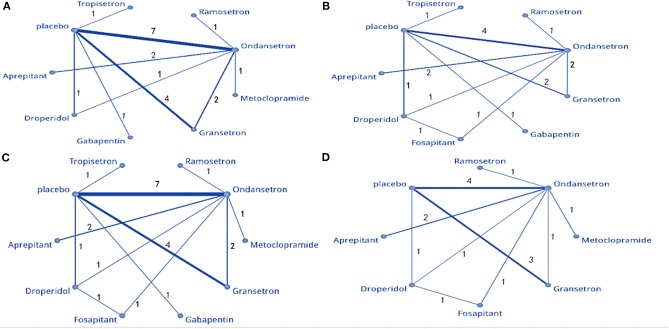
Network meta-analysis plot: **(A)** PON, **(B)** POV at 0–2 h, **(C)** POV at 0–24 h, and **(D)** POV at 0–48 h.

**Figure 4:**
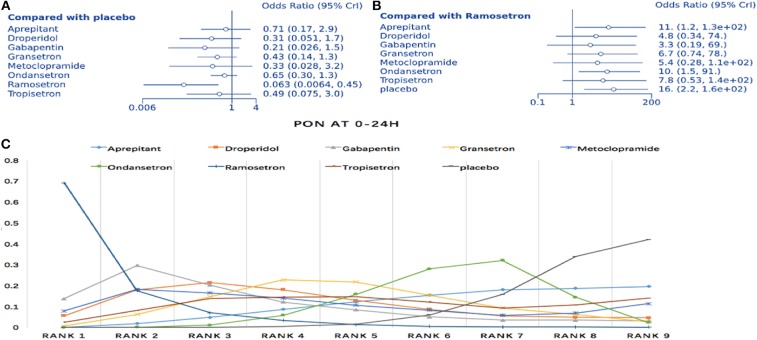
Analysis of PON. **(A)** Forest plot through using placebo as a comparator. **(B)** Forest plot using ramosetron as a comparator. **(C)** SUCRA plot.

The NMA involved direct and indirect comparisons amongst limited interventions for PON during the first 24 h post-surgery. A total of 15 trials were included, comparing ten interventions (1,183 participants; Figure 3A). Compared to placebo, significantly lower PON was only evident in patients receiving ramosetron (OR: 0.063, 95% Crl: 0.006–0.45; Figure 4A). When compared to ramosetron, three interventions including aprepitant, ondansetron and placebo showed significantly higher PON rates (Figure 4B). Based on SUCRA results, ramosetron showed a 69.2% probability as the best treatment option for POV during the first 24 h (Figure 4C). Gabapentin was ranked second, with a 29.7% probability, while droperidol (21.5%), and gabapentin (20.3%) had similar probability as third best treatment. Consistency assessment did not reveal significant differences (*P* = 0.839, Appendix 2A). Assessment of heterogeneity found *I*^2^ = 0% for granisetron vs. placebo, ondansetron vs. placebo, and aprepitant vs. ondansetron; as well as *I*^2^ = 27% for granisetron vs. ondansetron (Appendix 3A). Sensitivity analysis was performed omitting trials with a high risk of bias and lacking dexamethasone treatment. No differences were observed in comparison to the primary analysis.

### Anti-emetic Effectiveness for Postoperative Vomiting

#### Prophylaxis for Postoperative Vomiting During the First 2 h

**Figure 5:**
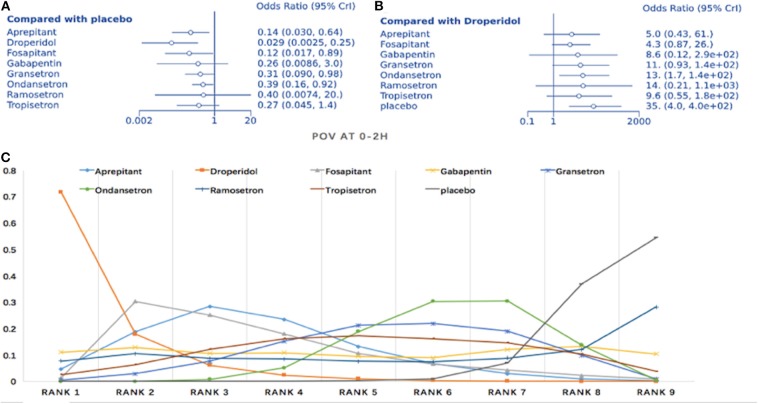
Analysis of POV at 0–2 h. **(A)** Forest plot, with placebo as comparator. **(B)** Forest plot, with droperidol as comparator. **(C)** SUCRA plot.

The NMA included 12 trials comparing nine interventions (1,074 total participants; Figure 3B). Compared to placebo, all treatments except gabapentin, ramosetron, and tropisetron displayed significant differences regarding POV treatment (droperidol: OR = 0.029, 95%, Crl: 0.003–0.25; Figure 5A). When droperidol was set as the baseline treatment, only ondansetron and placebo displayed a significantly poorer treatment effect for POV (Figure 5B). Regarding the probability ranking, we observed a 71.7% likelihood for droperidol as the best prophylaxis option for POV during the first 2 h. Fosaprepitant showed the second-highest probability with 30.3% (Figure 5C). No significant differences were observed between direct and indirect comparisons regarding consistency (*P* > 0.05, Appendix 2B). Heterogeneity assessment found *I*^2^ = 0% (Appendix 3B). Sensitivity analysis was performed by omitting studies with a high risk of bias, as well as studies that did not use dexamethasone. Differences were observed in droperidol vs. placebo (OR = 0.11, 95% Crl: 0.003–1.1) and fosaprepitant vs. placebo (OR = 0.9, 95% Crl: 0.017–25) (Appendix 4).

#### Prophylaxis for Postoperative Vomiting During the First 24 h

**Figure 6:**
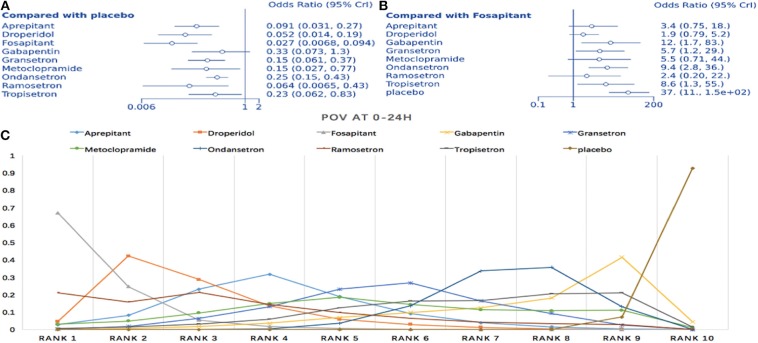
Analysis of POV at 0–24 h. **(A)** Forest plot with placebo as a comparator. **(B)** Forest plot with fosaprepitant as comparator. **(C)** SUCRA plot.

Seventeen articles containing 10 different interventions were included in this NMA (1,433 patients; Figure 3C). Compared to placebo, all treatments except gabapentin reduced POV during the first 24 h (fosaprepitant: OR = 0.027, 95% Crl: 0.007–0.093; Figure 6A). When considering fosaprepitant as the baseline treatment, gabapentin, granisetron, ondansetron, tropisetron, and placebo were associated with a higher POV incidence between 0 and 24 h post-surgery (Figure 6B). Based on SUCRA, we observed a 66.9% probability for fosaprepitant as the best treatment for POV during the first 24 h, followed by droperidol (42.2% probability) (Figure 6C). Consistency was assessed via direct and indirect comparisons, with no differences; *P* > 0.05 (Appendix 2C). In heterogeneity assessment, all four direct comparisons showed *I*^2^ = 0% (Appendix 3C). Sensitivity analysis was assessed omitting high-risk trials, without statistically significant differences.

#### Prophylaxis for Postoperative Vomiting During the First 48 h

**Figure 7:**
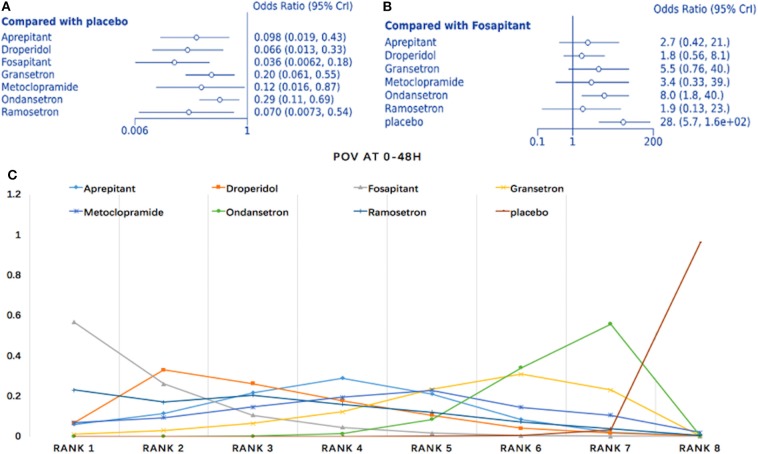
Analysis of POV at 0–48 h. **(A)** Forest plot using placebo as comparator. **(B)** Forest plot using fosaprepitant as comparator. **(C)** SUCRA plot.

Twelve studies comparing eight interventions were included in this NMA (911 total participants; Figure 3D). Compared to placebo, all treatments were associated with significantly improved effects on POV (fosaprepitant: OR = 0.036, 95% Crl: 0.006–0.18; Figure 7A). When fosaprepitant was set as the baseline treatment, only ondansetron and placebo administration displayed a significantly higher POV incidence during the first 48 h (ondansetron: OR = 8.0, 95% Crl: 1.8–40; Figure 7B). According to the probability ranking, fosaprepitant showed a 56.6% likelihood for being the most effective POV treatment during the first 48 h, followed by droperidol (33% probability; Figure 7C). Consistency assessment found no differences (*P* > 0.05; Appendix 2D). Heterogeneity assessment showed *I*^2^ = 0%, excluding ondansetron vs. placebo (*I*^2^ = 39%; Appendix 3D). Sensitivity analysis was performed omitting high-risk studies and no statistically significant differences were observed.

### Requirement for Rescue Anti-emetic Drugs

**Figure 8:**
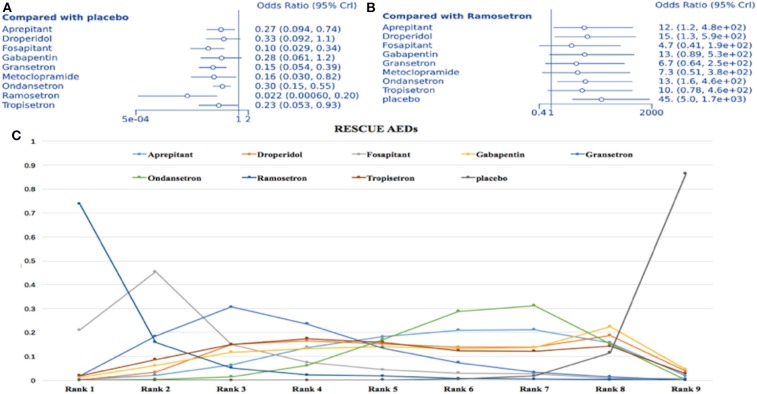
Analysis of rescue AED requirement. **(A)** Forest plot, with placebo as comparator. **(B)** Forest plot, with ramosetron as comparator. **(C)** SUCRA plot.

Fifteen studies with 10 treatment methods were included (1,293 participants). Compared to placebo, all treatments except droperidol and gabapentin were associated with a lower rescue antiemetic requirement (ramosetron: OR = 0.022, 95% Crl: 0.001–0.2; Figure 8A). Considering ramosetron as the baseline treatment, aprepitant, droperidol, ondansetron, and placebo were associated with a higher rate of requirement for rescue AED (Figure 8B). SUCRA plots showed a strong likelihood (83.5%) for ramosetron having the lowest rate of rescue AED requirement (Figure 8C). Heterogeneity assessments showed *I*^2^ = 0% for aprepitant vs. ondansetron, granisetron vs. ondansetron, and granisetron vs. placebo. On the other hand, *I*^2^ = 2% was recorded for ondansetron vs. placebo. In sensitivity analysis, no results were statistically different after omitting one trial with a high risk of bias and two single therapy trials.

### Complete Responses

For assessment of complete responses, seven trials and eight interventions were included (612 participants). Compared to placebo, only ramosetron showed a significantly higher incidence of complete responses (OR = 29, 95% Crl: 1.4–6.5e + 02). SUCRA analysis revealed a 57.8% probability for ramosetron as the best AED for PONV during the first 24 h (Appendix 5).

### Adverse Events

#### Headache

Two trials and four interventions were included (191 participants). Compared to placebo, the incidence of headache following AED treatment did not change (granisetron: OR = 0.63, 95% Crl: 0.06–4.5; Appendix 6A). We observed a 42.8% probability that granisetron was associated with the lowest incidence of headache (Appendix 6B).

#### Sedation

Three trials with three different interventions were included (267 participants). No differences were evident for granisetron or ondansetron when compared to placebo. Probability ranking showed that granisetron was associated with the lowest rate of excessive sedation (57.1%). Of the four interventions, ondansetron was most likely to be associated with excessive sedation (88.1%) (Appendix 7).

## Discussion

We performed a systematic review to evaluate the efficiency of different AED for PONV after craniotomy. Subsequently, we conducted an NMA to assess the effectiveness of intervention options for PONV prophylaxis. We established several novel findings. Firstly, ramosetron was associated with an improved prophylactic effect compared to other medications, with a 69.2% probability of being the best prophylaxis for PON during the first 24 h. In addition, ramosetron was more effective for PON than aprepitant, ondansetron, and placebo during the first 24 h. Ramosetron was also associated with the highest incidence of complete response and had the lowest rate of rescue AED requirement. These results are consistent with a previous meta-analysis in which ramosetron was found to be more effective for PONV with fewer side effects in comparison with ondansetron. Aprepitant showed no statistically significant differences in reducing PON incidence (25).

Secondly, fosaprepitant appeared to be the most effective prophylactic medication for POV, both between 0–24 and 0–48 h postoperatively. Droperidol was the second most effective AED for reducing POV during this time period. Fosaprepitant (130 mg) was also more effective than ondansetron (4 mg) for POV prophylaxis in high-risk patients, both between 0–24 and 0–48 h postoperatively. A recent retrospective analysis presented similar results, with fosaprepitant displaying a greater prophylactic effect than ondansetron for POV at 0–2, 0–24, and 0–48 h post-surgery in patients with moderate to high PONV risk (26). The guidelines issued in 2014 recommend ondansetron (4 mg) in combination with dexamethasone (4 mg) for PONV prophylaxis in high-risk patients (5). In addition, aprepitant has been shown to be superior to ondansetron for the prevention of vomiting. This effect of aprepitant on nausea and vomiting may be attributed to its differential impact on the pathophysiology of these phenomena. NK-1R antagonists provide antiemetic effects mainly via central regulation of visceral function, which can be achieved by suppressing neuronal activity at the nucleus of the solitary tract (NST), which controls the vomiting reflex (27).

Thirdly, droperidol was found to be the most effective AED for POV during the 0–2 h postoperative interval and was the second-best option between both 0–24 and 0–48 h. This is consistent with previous studies reporting low-dose droperidol to be effective for PONV prophylaxis (28).

Our findings provide new insights into the most beneficial AED treatment regimens. Firstly, combination therapy is advised for high-risk patients. Because perioperative dexamethasone was used in the majority of the included studies, these treatments should be implemented in combination with dexamethasone. Secondly, only one included trial studied ramosetron, where it was demonstrated that 0.3 mg IV administration was effective for PONV prophylaxis and found a reduced antiemetic requirement, which is consistent with a previous study (29); thus, further trails on this drug are necessary. Thirdly, in this NMA, we included a small number of trials without dexamethasone usage. Therefore, it is unclear whether fosaprepitant in combination with dexamethasone will produce additional benefits compared to fosaprepitant alone, although current guidelines recommend combination therapy over single therapy. Furthermore, we only analyzed PONV during the first postoperative day, while previous research suggests nearly 30% of patients still have PONV up to 3 days postoperatively. Finally, apart from corticosteroids, the mechanism of action of AED involves the antagonism of neurotransmitter receptors in the gastrointestinal tract and the central nervous system. Chemotherapy-induced nausea and vomiting (CINV) has similar traits to PONV (27). Previous studies have proven the antiemetic effects of NK-1R antagonists in patients undergoing moderately to highly emetogenic chemotherapy, as well as in different types of tumor-related surgery (30), so our findings may provide new insight in the management of CINV and other surgery-related PONV.

The disparate results of our sensitivity analysis between droperidol vs. placebo and fosaprepitant vs. placebo with respect to POV at 0–2 h post-surgery may be due to insufficient study data and the heterogeneity of perioperative dexamethasone usage (4, 5). Differences in the results of droperidol vs. placebo may also be accounted for by the different administration times and dosages assessed in the included trials. For example, droperidol may the most effective AED for PONV prophylaxis when administrated at the end of surgery (5).

This systematic review and network meta-analysis were not without limitations. Firstly, only 16 trials with nine AED were included and more relevant RCT are needed. Secondly, dexamethasone was only regarded as an adjuvant therapy and was not included in our AED data analysis. Nevertheless, it has been reported to have an antiemetic effect comparable to ondansetron (31). We were unable to conduct an NMA to separate trials omitting dexamethasone usage due to the lack of currently available data. One trial with a high risk of bias was also included, and as such, we performed sensitivity analysis for all outcomes, which focused on omitting trials without perioperative dexamethasone and a high risk of bias. Further trials assessing AED efficacy with and without dexamethasone are required in future studies. Thirdly, we were unable to explore other risk factors with a proven relationship to PONV, including surgery site, duration of anesthesia, and the use of volatile anesthetics and nitric oxide (4), due to inconsistencies in data reporting and the small number of studies included. Finally, the included trials contain differences that could potentially influence outcomes. Propofol has been reported to have anti-emetic properties (5, 32), and propofol combined with volatile anesthesia is related to lower PON incidence (33). Some data from the included studies used propofol as an anesthetic method. In addition, the intracranial procedure was not specified in 70% of the studies included; this is important, given the known link of these procedures to PONV incidence (34)31. Thus, further RCT using a larger number of participants are required to support our conclusions.

## Conclusions

In this systematic review and NMA on patients who underwent craniotomy, direct, and indirect comparisons revealed ramosetron as the best AED for PON during the first 24 h. Ramosetron was also associated with the lowest incidence of PONV and displayed the lowest requirement for rescue AED. Fosaprepitant was the best AED for reducing POV on the first and second postoperative days, with droperidol representing the next best alternative. Furthermore, fosaprepitant was more effective than ondansetron for POV prophylaxis during the first and second postoperative days.

These findings provide practical and supportive evidence for future clinical treatment guidelines and research on PONV following craniotomy. They also offer insight regarding the management of CINV and other surgery-related PONV to enhance patients' recovery and improve their quality of life.

## Data Availability Statement

The raw data supporting the conclusions of this article will be made available by the authors, without undue reservation, to any qualified researcher.

## Author Contributions

YC and JC: conceptualization, validation, and data curation. YC: methodology and software. JC: resource and supervision. JC: writing-original draft and writing- review and editing.

### Conflict of Interest

The authors declare that the research was conducted in the absence of any commercial or financial relationships that could be construed as a potential conflict of interest.
