# Supplementary material for: Anti-emetic Drugs for Prophylaxis of Postoperative Nausea and Vomiting After Craniotomy: An Updated Systematic Review and Network Meta-Analysis
Source: Front Med (Lausanne). 2020 Feb 25;7:40. doi: 10.3389/fmed.2020.00040 (PMC7052291; doi:10.3389/fmed.2020.00040)

SUPPLYMENTARY DATA

**Appendix 1:** Disposition after full text review

| Primary author | Journal | Year | Decision |
| --- | --- | --- | --- |
| Ryu J H | BMC Anesthesiol | 2014 | include |
| Atsuta J | J Anesth | 2017 | include |
| Fabling, J. M. | Anesth Analg | 2000 | include |
| Fabling, J. M. | J Neurosurg Anesthesiol | 2002 | include |
| Eberhart L H J | Best Pract Res Clin Anaesthesiol | 2007 | Exclude-not RCT |
| Gan, T. J. | Anesth Analg | 2014 | Exclude- guideline |
| Habib A S. | Anesth Analg | 2011 | Include |
| Irefin S A. | J Anesth | 2003 | Exclude-not RCT |
| Jain V. | J Neurosurg Anesthesiol | 2009 | Include |
| Jellish W S. | Otolaryngol Head Neck Surg | 2006 | Exclude -not primary outcome |
| Kathirvel, S. | J Neurosurg Anesthesiol | 2001 | Include |
| Lonjaret, L | Anaesth Crit Care Pain Med | 2017 | Exclude-no prophylaxis AEDs |
| Lv J. Q. | Trials | 2013 | Exclude -not AEDs |
| Lu, J. Q. | Zhen Ci Yan Jiu | 2013 | Exclude-not AEDs |
| Madenoglu H. | J Neurosurg Anesthesiol | 2003 | Include |
| Misra S. | J Neurosurg Anesthesiol | 2013 | Include |
| Nilsson I. | J Neurosurg Anesthesiol | 2015 | Exclude -not AEDs |
| Ouyang M.W. | Curr Med Res Opin | 2013 | Exclude-not RCT |
| Ouyang M.W | Curr Med Res Opin | 2013 | Exclude-not RCT |
| Burkhardt T | J Neurosurg Anesthesiol | 2014 | Exclude-not RCT |
| Singh PM | J Clin Anesth | 2016 | Exclude-not RCT |
| Soliman R N. | Middle East J Anaesthesiol | 2011 | Exclude-not PONV |
| Hartsell T. | Anesth Analg | 2005 | Exclude-no prophylaxis AEDs |
| Tsutsumi Y. M. | Biomed Res Int | 2014 | Include |
| Wang X. Q. | J Neurosurg Anesthesiol | 2010 | Exclude -not AEDs |
| Wang Y. J. | Hunan Yi Ke Da Xue Xue Bao | 2002 | Include |
| Wig J. | J Neurosurg Anesthesiol | 2007 | Include |
| Xu M. | J Neurosurg Anesthesiol | 2012 | Exclude -not AEDs |
| Manninen PH | J Clin Anesth | 2002 | Exclude -not AEDs |
| Fabling, J. M. | J Neurosurg Anesthesiol | 1997 | Exclude-not RCT |
| Hellickson J. D. | J Neurosci Nurs | 2016 | Exclude -no AEDs |
| Latz B. | J Neurosurg | 2011 | Exclude -no AEDs |
| Meng, L | J Neurosurg Anesthesiol | 2006 | Exclude -no AEDs |
| Sato K. | J Anesth | 2013 | Exclude -not RCT |
| Tan C | J Neurosurg Anesthesiol | 2012 | Exclude -not RCT |
| Uribe A | Anesthesia and analgesia | 2011 | Exclude –meeting report |
| Ilhan O | Eurasian J Med. | 2010 | Exclude -not primary outcome |
| Flynn B. C. | Anesth Analg | 2006 | Exclude -not RCT |
| Neufeld SM | J Neurosurg Anesthesiol | 2007 | Exclude -not RCT |
| Gupta P | Anesth Essays Res | 2014 | Include |
| Kim MS, | Yonsei Med J | 2017 | Exclude -not RCT |
| Ahn E | PLoS One | 2016 | Exclude -not RCT |
| Kurita N | Masui | 2004 | Exclude -not RCT |
| Sha T | Forsch Komplementmed | 2015 | Exclude-not AEDs |
| Tsaousi GG | Curr Opin Anaesthesiol | 2017 | Exclude -not RCT |
| Tanskanen PE | Br J Anaesth | 2006 | Exclude-not PONV |
| Chatellier A | Rev Stomatol Chir Maxillofac | 2012 | Exclude-not AEDs |
| Audibert G | Ann Fr Anesth Reanim | 2004 | Exclude -not RCT |
| Kurita N | Masui | 2004 | Exclude -not RCT |
| Tseĭtlin AM | Anesteziol Reanimatol | 2003 | Exclude-not adult |
| Bergese SD | Front Med (Lausanne) | 2016 | Include |
| Heidari S.M | Journal of Isfahan Medical School | 2012 | Exclude-not AEDs |
| Habib A.S. | Anesthesia and Analgesia | 2004 | Exclude-not craniotomy |
| Najeeb R | JK Practitioner | 2000 | Exclude-meeting report |
| Fujii Y | Anesthesia and Analgesia | 1998 | Exclude-not craniotomy |
| Dalal P | J Neurosurg Anesthesiol | 2012 | Exclude-duplicate |
| SergioD.Bergese | [Front Med (Lausanne)](https://www.ncbi.nlm.nih.gov/pmc/articles/PMC4469110/). | 2015 | Exclude -not RCT |
| Antor M A | Anesthesia and Analgesia | 2011 | Exclude -not RCT |
| Jolly C | Pharmacien Hospitalier et Clinicien | 2016 | Exclude-not AEDs |
| Connell J. | J Neurosurg Anesthesiol | 2010 | Exclude-different research design |
| Alon E | Der anaesthesist | 1994 | Exclude-not craniotomy |
| Urias E | Reg Anesth Pain Med | 2016 | Exclude- not craniotomy |
| Gandhi R | J Anaesthesiol Clin Pharmacol | 2009 | Exclude-not craniotomy |
| Wallenborn J | BMJ (clinical research ed.) | 2006 | Exclude-different research design |
| Lee S | Biomed research international | 2015 | Exclude-different research design |
| Klenke S | Br J anaesth | 2018 | Exclude-different research design |
| Birmingham SD | Military medicine | 2006 | Exclude-different research design |
| Lee S | Biomed res int | 2015 | Exclude-different research design |
| Bopp EJ | AANA journal | 2010 | Exclude-not craniotomy |
| Kleine Brueggeney M | Anesthesia and analgesia | 2015 | Exclude-not AEDs |
| Mayeur C | Ann Fr Anesth Reanim | 2012 | Exclude- not craniotomy |
| Levin DN | Can J Anaesth | 2017 | Exclude-not AEDs |
| Muñoz HR | Revista medica de chile, | 2006 | Exclude-different research design |
| Chun HR | British journal of anaesthesia | 2014 | Exclude- not craniotomy |
| Agarkar S | Indian journal of anaesthesia | 2015 | Exclude- not craniotomy |
| Yazbeck-Karam VG | Anesthesia and analgesia | 2017 | Exclude-different research design |
| Philip BK | Eur J anaesthesiol | 2000 | Exclude- not craniotomy |
| Kappen TH | Anesthesiology | 2014 | Exclude-different research design |
| Jones S | AANA journal | 2006 | Exclude- not craniotomy |
| Yong-Hun Lee | Br J Clin Pharmacol | 2016 | Exclude- not craniotomy |
| Senthil Kumar S | J Anaesthesiol Clin Pharmacol | 2007 | Exclude- not craniotomy |
| Sinha PK | J Neurosurg Anesthesiol | 1999 | Include |
| Pugh SC | Anaesthesia | 1996 | Include |
| Adel M El Shobaki | EgJ Anaesth | 2003 | Include |

**Appendix 2:** Consistency Assessment


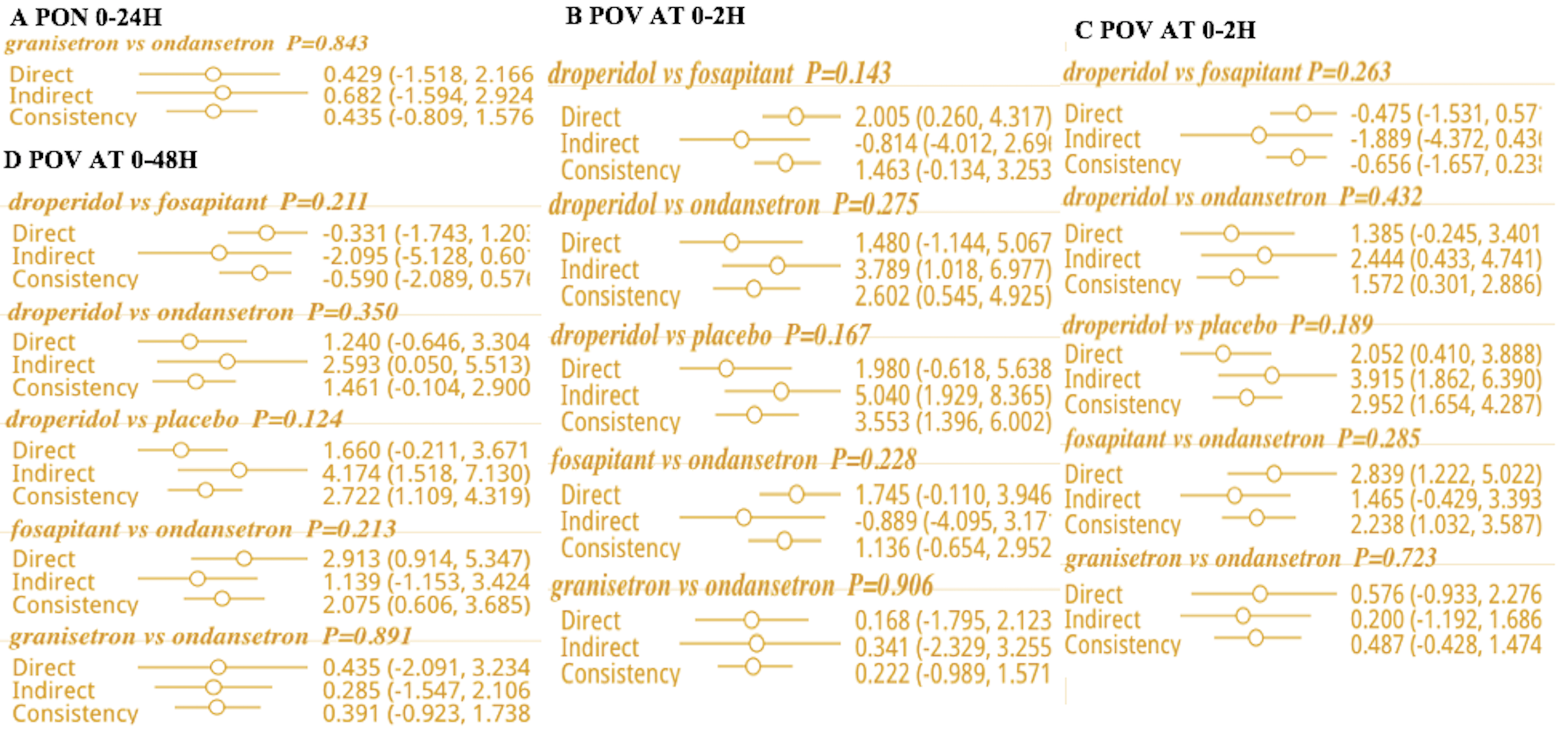


**Appendix 3:** Heterogeneity Assessment

A: PON AT 0-24H


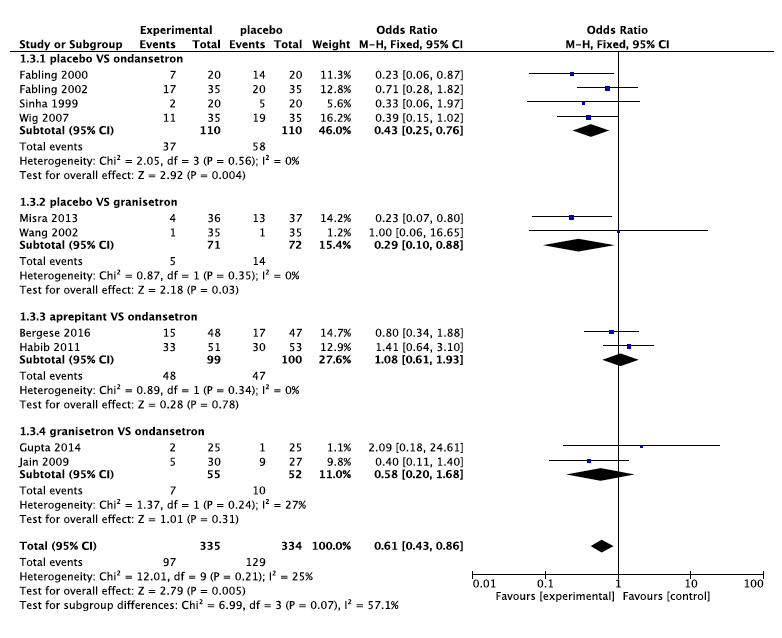


**B: POV AT 0-2H**

**
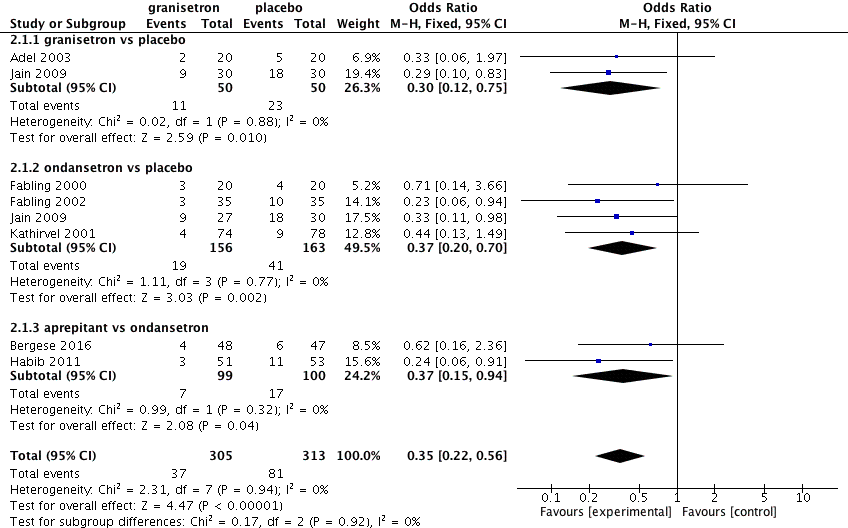
**

**C: POV AT 0-24H**

**
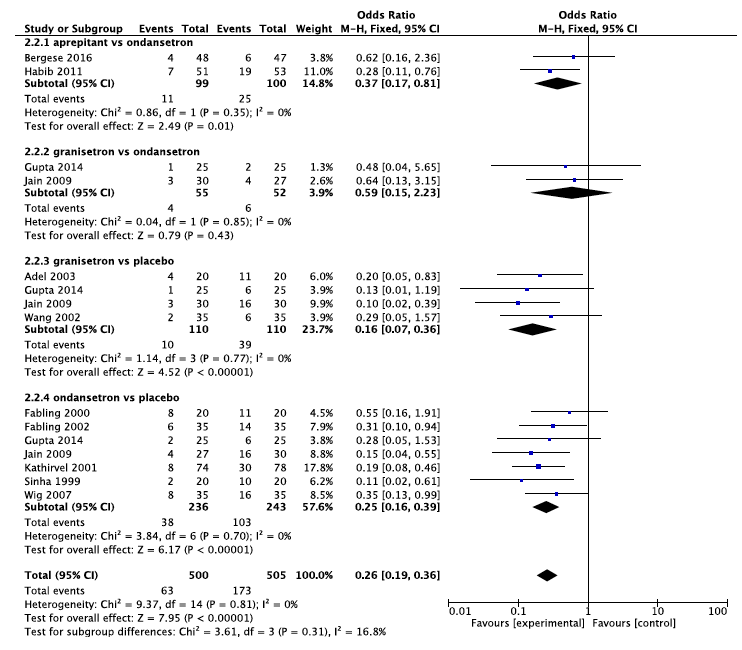
**

**D: POV AT 0-48H**

**
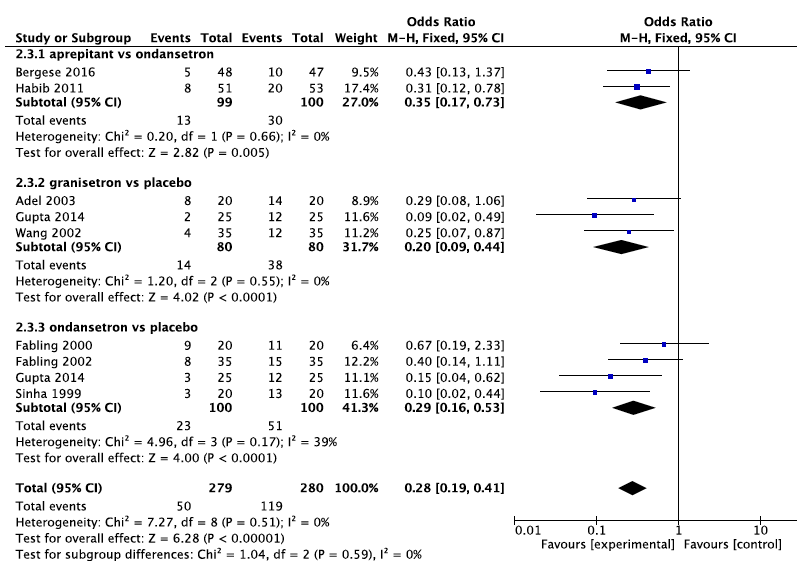
**

**Appendix 4: Sensitivity analysis (POV AT 0-2h)**

**
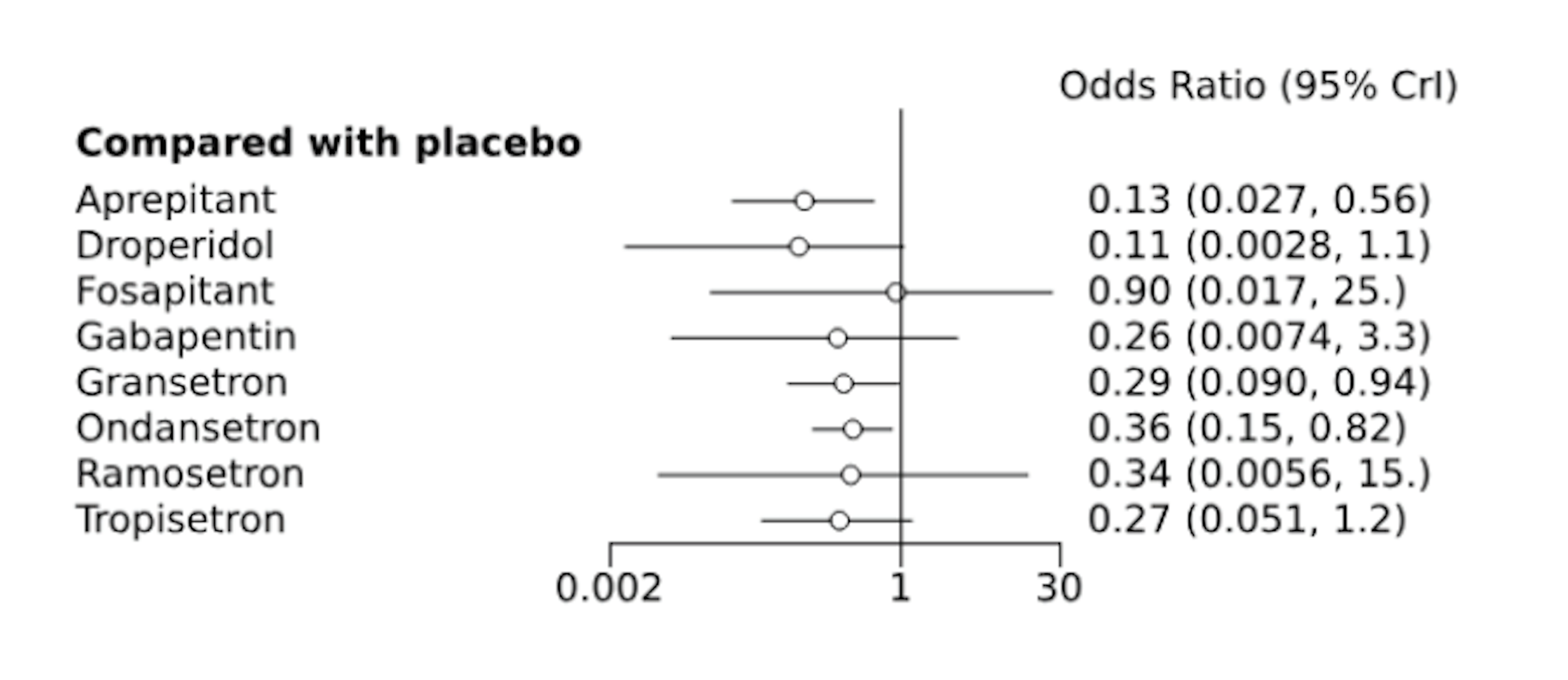
**

**Appendix 5: Analysis of complete response.** (A) Forest plot using placebo as comparator, (B) SUCRA plot.

**
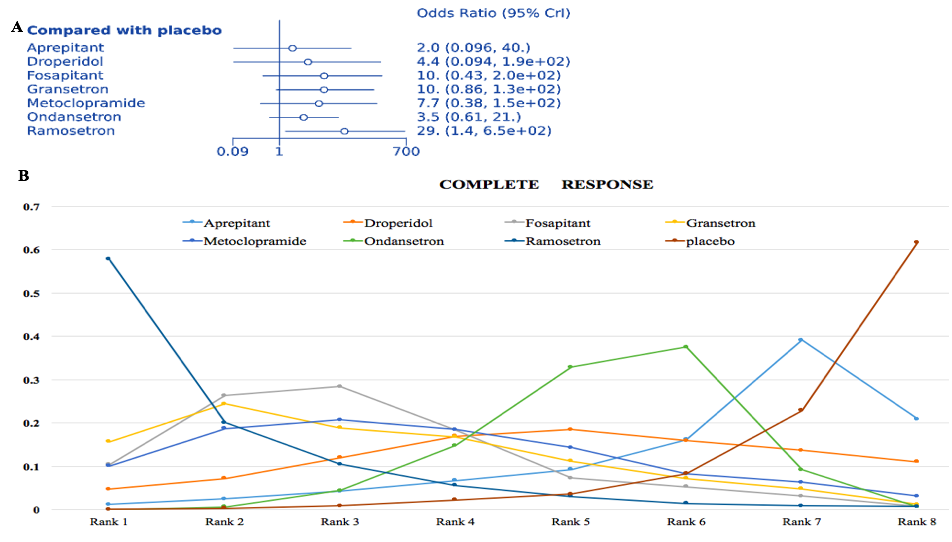
**

**Appendix 6: Analysis of headache**(A) Forest plot using placebo as comparator, (B) SUCRA plot.


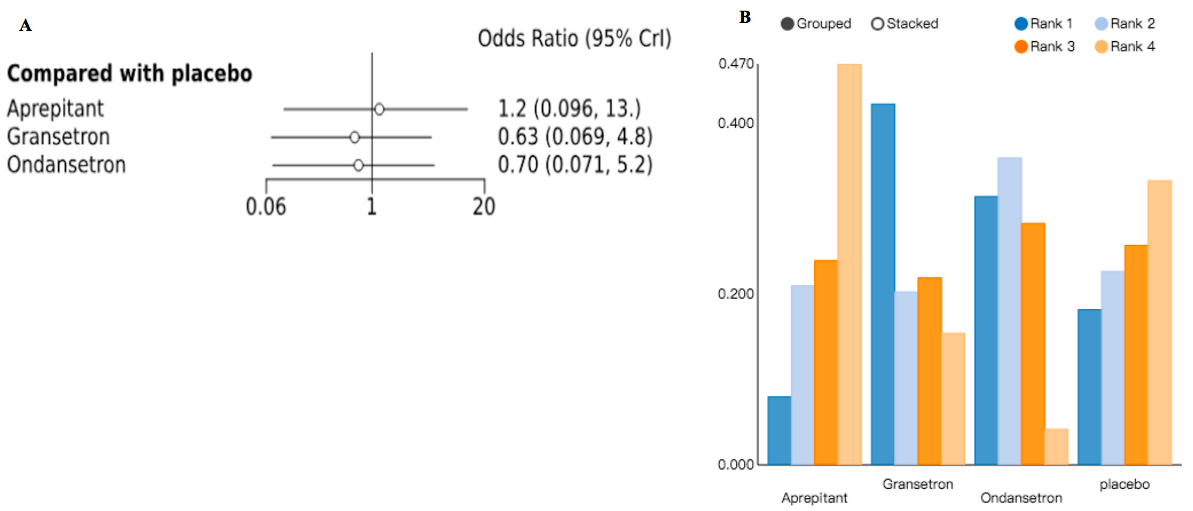


**Appendix 7: Analysis of Sedation** SUCRA plot.


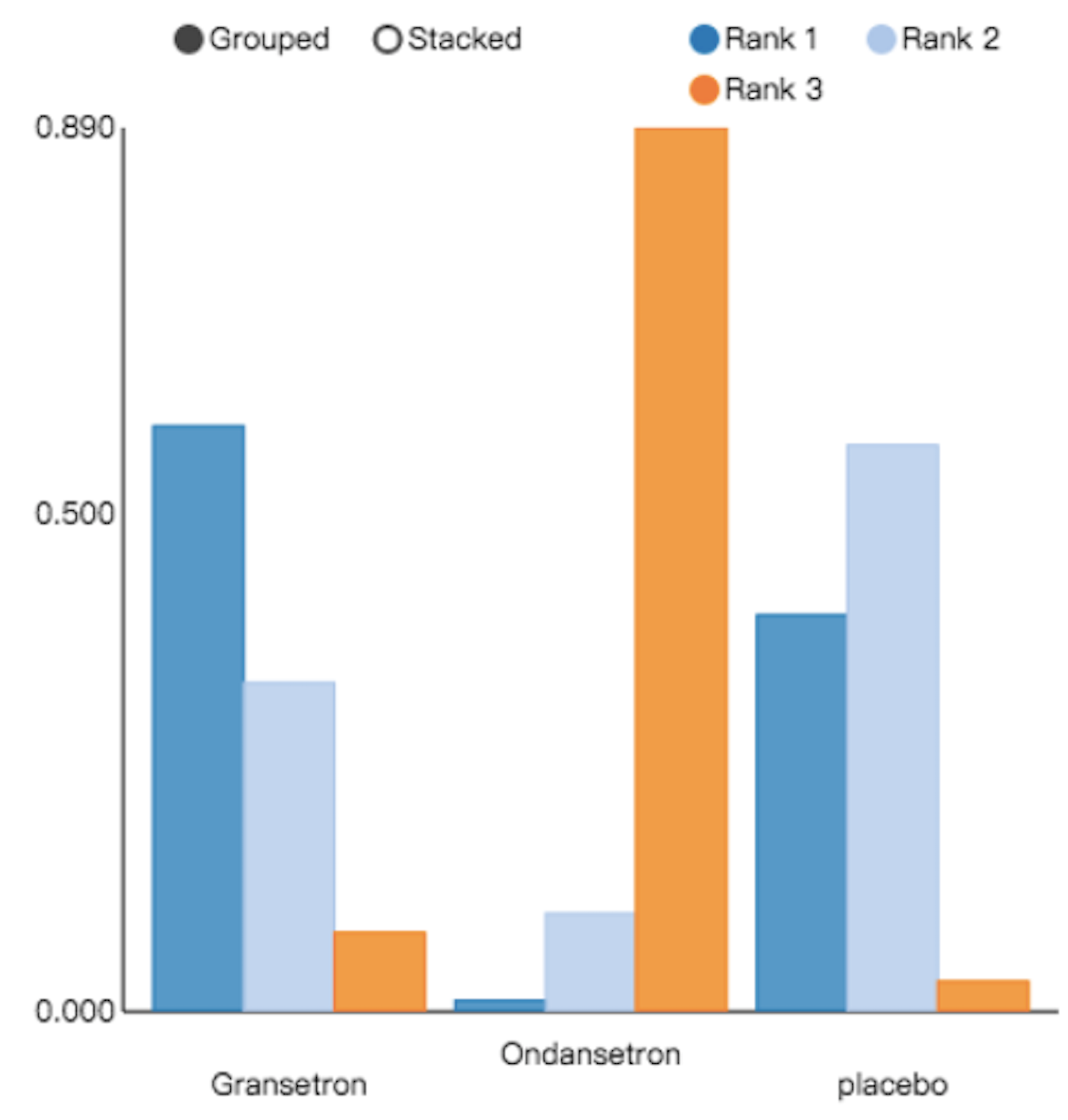

Supplement: Supplementary file 1 [file Data_Sheet_1.docx]
